# Early risk prediction model for intra-abdominal hypertension in adult ICU patients: a prospective cohort study

**DOI:** 10.3389/fmed.2026.1835766

**Published:** 2026-05-13

**Authors:** Yanrui Tan, Yujia Kou, Jiao Du, Yuqin Chen, Yi Ma, Hong Yu, Shasha Li, Chuanliang Pan

**Affiliations:** 1 Chengdu Third People's Hospital, Chengdu, China; 2 North Sichuan Medical College, Nanchong, China; 3 Affiliated Hospital of North Sichuan Medical College, Nanchong, China

**Keywords:** cox regression, intensive care unit, intra-abdominal hypertension, intra-abdominal pressure, nomogram, nursing assessment, risk prediction

## Abstract

**Objective:**

To identify independent risk factors for intra-abdominal hypertension (IAH) in adult intensive care unit (ICU) patients, develop a predictive nomogram, and perform internal validation.

**Methods:**

This prospective cohort study enrolled 261 adult ICU patients from two tertiary hospitals in Sichuan Province, China, between August and December 2022, using convenience sampling. Patients with IAH at admission were excluded. A standardized questionnaire captured demographic, clinical, and laboratory data. Cox proportional hazards regression identified independent predictors of IAH within 1–6 days post-ICU admission. A nomogram was constructed and internally validated using Bootstrap resampling (1,000 iterations). Model performance was assessed by the concordance index (C-index), area under the time-dependent receiver operating characteristic curve (AUC), calibration plots, and decision curve analysis (DCA).

**Results:**

Intra-abdominal hypertension occurred in 198 patients (75.9%), with a median onset of 12 h (range: 12–72 h). Multivariable Cox analysis revealed six independent risk factors: pre-ICU surgery (HR = 1.524, 95%CI: 1.075–2.161), abdominal distension (HR = 2.082, 95%CI: 1.505–2.881), increased intra-abdominal contents (HR = 1.857, 95%CI: 1.343–2.567), intraperitoneal fluid accumulation (HR = 1.500, 95%CI: 1.074–2.095), shock (HR = 1.806, 95%CI: 1.218–2.677), and APACHE II score (HR = 1.047 per point, 95%CI: 1.016–1.078). The nomogram demonstrated excellent discrimination (C-index: 0.826; bootstrap-corrected: 0.826) and calibration. Time-dependent AUC at 12 h, 24 h, and 36 h were 0.836 (95% CI: 0.787–0.884), 0.932 (95% CI: 0.900–0.964), and 0.913 (95% CI: 0.877–0.950), respectively. DCA confirmed clinical utility across a range of threshold probabilities.

**Conclusion:**

This nomogram, based on six readily available clinical variables, enables early identification of ICU patients at high risk for IAH, facilitating targeted monitoring and preventive interventions.

**Relevance to clinical practice:**

The model provides a simple bedside tool for risk stratification, guiding proactive intra-abdominal pressure monitoring and individualized care plans.

## 1 Introduction

Intra-abdominal pressure (IAP) is the steady-state pressure concealed within the abdominal cavity, generated by the hydrostatic forces of visceral organs, vascular tone, and resistance from the abdominal wall and diaphragm (1, 2). Under normal conditions, IAP ranges from 0 to 5 mmHg in healthy adults, but it can be physiologically elevated in obesity or after meals (3). In critically ill patients, however, sustained or repeated pathological elevation of IAP defines intra-abdominal hypertension (IAH), a condition that occurs in up to 50% of ICU admissions and is independently associated with increased morbidity and mortality (4, 5).

The pathophysiological consequences of IAH extend beyond the abdominal compartment. Elevated IAP reduces venous return, compresses the vena cava, and impairs cardiac output, leading to hemodynamic instability (6). It also decreases thoracic compliance, increases intrathoracic pressure, and exacerbates ventilation-perfusion mismatch (7). Renal perfusion is particularly vulnerable; IAP > 15 mmHg can reduce renal blood flow and glomerular filtration rate, contributing to acute kidney injury (8). Splanchnic hypoperfusion may result in bacterial translocation and systemic inflammatory response syndrome (9). If unrecognized or untreated, IAH can rapidly progress to abdominal compartment syndrome (ACS), a life-threatening condition with multi-organ failure (10).

Early detection of IAH is therefore paramount. The gold standard for IAP measurement is the intravesical technique, using a Foley catheter and a pressure transducer (5, 11). Although minimally invasive, this method carries risks of urinary tract infection, requires specific training, and is contraindicated in patients with bladder trauma, pelvic fractures, or neurogenic bladder (12). Moreover, universal screening of all ICU admissions is neither practical nor cost-effective. Alternative approaches, such as clinical examination (e.g., abdominal distension, firmness) or surrogate markers (e.g., elevated peak airway pressure), have low sensitivity and specificity (13).

Several scoring systems, such as the Acute Physiology and Chronic Health Evaluation II (APACHE II) and the Sequential Organ Failure Assessment (SOFA), have been correlated with IAH, but they were designed to assess disease severity and organ dysfunction, not to predict IAH specifically (14). Consequently, there is a pressing need for a simple, non-invasive, and accurate tool that can identify patients at high risk for IAH early in their ICU course.

In response to this gap, we conducted a prospective cohort study to identify independent risk factors for IAH in adult ICU patients and to develop a predictive nomogram. A nomogram is a graphical representation of a statistical model that translates complex regression equations into a user-friendly interface, allowing clinicians to estimate individual probabilities by summing points assigned to each predictor (15). By integrating readily available clinical variables, such a tool could empower bedside nurses and physicians to prioritize monitoring and implement preventive strategies before IAH becomes established.

## 2 Methods

### 2.1 Study design and population

This prospective cohort study was conducted in the intensive care units of two tertiary hospitals in Sichuan Province, China, from August 20, 2022, to December 15, 2022. Patients were enrolled using a convenience sampling method.

**Figure 1:**
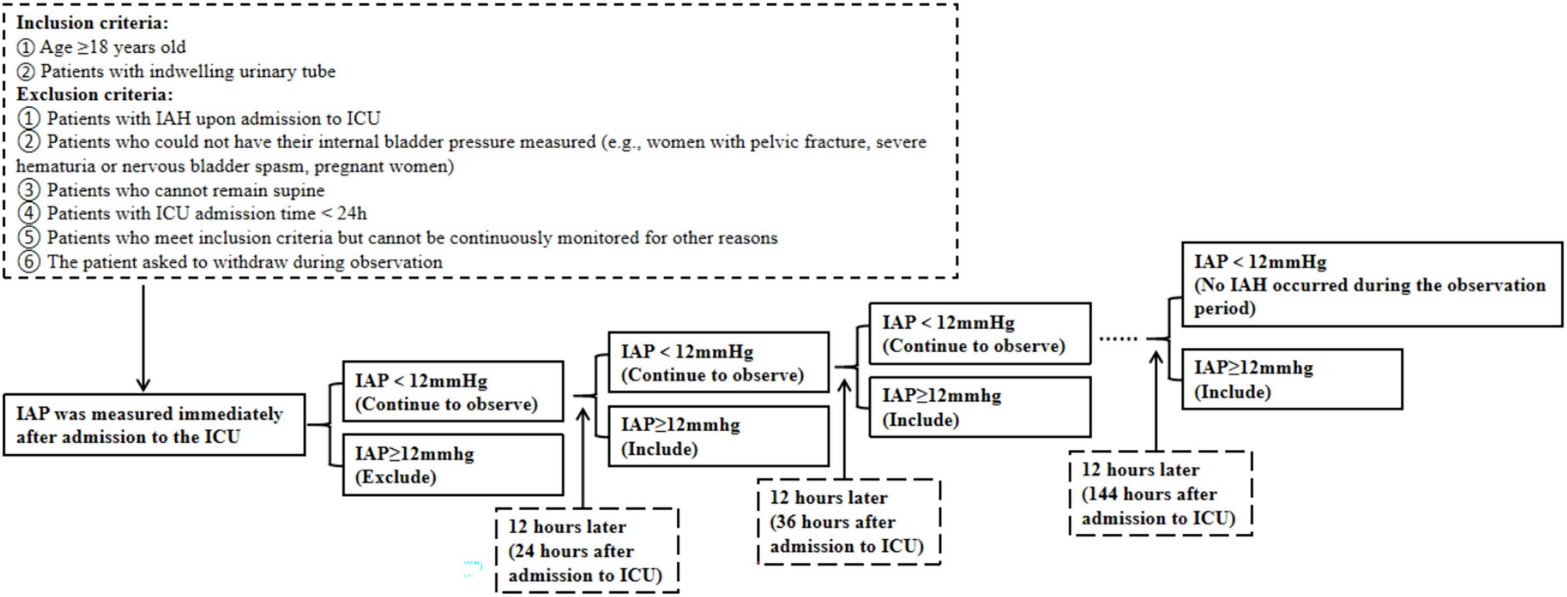
Flowchart of patient inclusion process.

Inclusion criteria were: (1) age ≥18 years; (2) presence of an indwelling urinary catheter. Exclusion criteria were: (1) IAH (IAP ≥ 12 mmHg) at the time of ICU admission; (2) contraindications to intravesical pressure measurement (pelvic fracture, severe hematuria, neurogenic bladder, pregnancy); (3) inability to maintain a supine position; (4) expected ICU stay <24 h. Patients were also excluded if they met any exclusion criterion during follow-up or if they withdrew consent. The inclusion and exclusion criteria for patients are shown in Figure 1.

The sample size was determined based on the “10 events per variable” (EPV) principle recommended for prediction model development (16–18). Assuming 5–7 candidate predictors in the final model and an expected IAH incidence of 30–40% in the ICU (2), we calculated a minimum requirement of 245 patients after accounting for a 5% loss to follow-up. The final model included six predictors, and the incidence of IAH was 75.9%. The 261 patients we enrolled met the sample size requirements for this study.

### 2.2 Data collection and variables

A standardized case report form was developed through an extensive literature review and expert consensus among intensivists, surgeons, and critical care nurses. Except for IAH, all the collected data were generated within 12 h after the patient was admitted to the ICU, which reflects the patient’s health condition within 12 h after admission to the intensive care unit. The form collected the following categories of data:

Demographic and baseline characteristics: age, sex, body mass index (BMI), and whether the patient underwent surgery immediately prior to ICU admission.

Clinical parameters at ICU admission and during follow-up: oliguria (urine output <0.5 mL/kg/h for ≥6 h), hypokalemia (serum potassium <3.5 mmol/L), abdominal distension defined as a sagittal abdominal diameter exceeding the anteroposterior diameter of the chest or pelvis on visual inspection, pneumonia, various types of shock (septic, hemorrhagic, cardiogenic, or hypovolemic), use of vasoactive drugs, positive end-expiratory pressure (PEEP) ≥ 7 cmH₂O (19), mean arterial pressure (MAP), central venous pressure (CVP), and laboratory values (lactate, urea, creatinine, uric acid).

Conditions related to IAH pathophysiology: increased intra-abdominal contents (gastroparesis, gastric dilatation, ileus, intestinal obstruction, pseudo-obstruction, abdominal or retroperitoneal tumors), intraperitoneal fluid accumulation (ascites due to cirrhosis, pancreatitis, or ovarian hyperstimulation; hemoperitoneum; pneumoperitoneum; peritoneal dialysis; inflammatory peritonitis), decreased abdominal wall compliance (obesity, abdominal surgery, prone positioning, abdominal wall hematoma, burns, high PEEP, patient-ventilator dyssynchrony), and capillary leak syndrome requiring fluid resuscitation.

Severity scores: APACHE II and SOFA scores calculated within the first 24 h of ICU admission.

Three trained research nurses, supervised by clinical specialists, performed all IAP measurements and data collection. A fourth researcher independently verified data accuracy. Inter-rater reliability was ensured through periodic calibration sessions.

Because this was a prospective study with standardized data collection protocols, the overall rate of missing data was minimal. Any candidate variable for which missing observations exceeded 20% was pre-specified for exclusion from the analysis. No variable retained in the final model exceeded this threshold, with all analyzed variables having less than 2% missing data. Accordingly, a complete-case analysis was performed without the need for imputation.

### 2.3 Outcome indicators and definition

The evaluation indicator for this result is to observe whether patients develop intra-abdominal hypertension (IAH) within 1 to 6 days after being admitted to the intensive care unit. The criteria for determining intra-abdominal hypertension are as follows: If the intra-abdominal pressure (IAP) is ≥12 mmHg (2, 5), it is considered as the occurrence of intra-abdominal hypertension. IAP was measured via the intravesical route with the patient in the supine position, at end-expiration, after ensuring zeroing at the mid-axillary line, and using 25 mL of sterile saline instillation (20).

### 2.4 Statistical analysis

Statistical analyses were performed using SPSS version 23.0 and R 4.2.2 with the rms and survival packages. Normally distributed continuous variables were expressed as mean ± standard deviation, non-normally distributed variables as median (interquartile range, IQR), and categorical variables as frequencies (percentages). For the prediction model, we first conducted univariable Cox proportional hazards regression to screen potential predictors associated with IAH, using a significance level of *p* < 0.05. Variables with *p* < 0.05 were entered into a multivariable Cox regression model with backward stepwise selection (likelihood ratio method) to identify independent predictors. A nomogram was constructed based on the final multivariable Cox model to predict IAH-free probability at 12, 24, and 36 h after ICU admission. For each predictor, a point scale was derived from the regression coefficients, and the total points were linked to the predicted survival probabilities. Internal validation was performed using Bootstrap resampling with 1,000 iterations to obtain bias-corrected estimates of model performance. Discrimination was assessed by the concordance index (C-index) and time-dependent area under the receiver operating characteristic curve (AUC). Calibration was evaluated by plotting the predicted versus observed IAH probabilities at 12, 24, and 36 h, using the loess smoother. Clinical utility was examined using decision curve analysis (DCA), which quantifies the net benefit of using the model to guide interventions across a range of threshold probabilities (21). All statistical tests were two-tailed, and a *p*-value < 0.05 was considered statistically significant.

### 2.5 Ethical considerations

This study was approved by the ethics committees of participating hospitals (File Number: 2022ER265-1). Written informed consent was obtained from all patients or their legal representatives prior to enrollment. The study was conducted in accordance with the Declaration of Helsinki.

## 3 Results

### 3.1 Patient characteristics and IAH incidence

A total of 261 ICU patients were included. The median age was 62 years, and 59% were male. The median BMI was 24.5 kg/m^2^. During the observation period, 198 patients developed IAH. The median time to IAH onset was 12 h after ICU admission. Most IAH events occurred within the first 36 h: 104 patients (52.5%) at 12 h, 60 patients (30.3%) between 12 and 24 h, and 23 patients (11.6%) between 24 and 36 h. Only 11 patients (5.6%) developed IAH after 36 h.

### 3.2 Univariable cox regression analysis

Univariable Cox regression identified 16 variables significantly associated with IAH (*p* < 0.05). These included: age, BMI, pre-ICU surgery, abdominal distension, increased intra-abdominal contents, Intraperitoneal fluid accumulation, decreased abdominal wall compliance, shock, use of vasoactive drugs, PEEP ≥7 cmH₂O, MAP, lactate, urea, uric acid, APACHE II score, and SOFA score. Sex, oliguria, hypokalemia, capillary leak/fluid resuscitation, pneumonia, and creatinine were not significant in univariable analysis. The specific situation is shown in Table 1.

**Table 1:** Univariate analysis of risk factors for IAH in ICU patients.

Factors	Regression coefficient (*β*)	Standard error (SE)	*P*	HR	95%CI
Age	0.010	0.004	0.010*	1.010	1.002–1.018
Gender (female)	0.251	0.144	0.083	0.778	0.587–1.033
Pre-ICU surgery (yes)	0.395	0.145	0.007*	1.484	1.117–1.972
BMI	0.001	0.000	0.001*	1.001	1.000–1.002
Oliguria (yes)	0.244	0.191	0.201	1.277	0.878–1.856
Hypokalemia (yes)	0.049	0.143	0.734	0.953	0.720–1.261
Abdominal distension (yes)	1.125	0.148	<0.001*	3.080	2.303–4.119
Increased abdominal cavity contents (yes)	0.623	0.147	<0.001*	1.865	1.398–2.486
Intraperitoneal fluid accumulation (yes)	0.706	0.148	<0.001*	2.026	1.516–2.708
Decreased abdominal wall compliance (yes)	0.388	0.150	0.010*	1.473	1.097–1.978
Capillary leak/fluid resuscitation (yes)	0.333	0.198	0.093	1.396	0.946–2.059
Pneumonia (yes)	0.277	0.149	0.063	1.320	0.984–1.768
Shock (yes)	0.820	0.145	<0.001*	2.269	1.709–3.013
Use of vasoactive drugs (yes)	0.615	0.145	<0.001*	1.850	1.392–2.458
PEEP ≥ 7cmH2O (yes)	0.323	0.144	0.025*	1.382	1.041–1.833
MAP	0.011	0.003	<0.001*	0.989	0.983–0.995
Lactic acid	0.056	0.014	<0.001*	1.058	1.030–1.087
Urea	0.007	0.002	<0.001*	1.007	1.003–1.011
Creatinine	0.000	0.000	0.492	1.000	0.999–1.000
Uric acid	0.001	0.000	0.001*	1.001	1.000–1.002
APACHE II	0.077	0.009	<0.001*	1.080	1.062–1.098
SOFA	0.121	0.015	<0.001*	1.128	1.097–1.161

^*^ means *p* < 0.05 (statistically significant).

### 3.3 Multivariable cox regression analysis

In the univariate analysis, the variables with statistical significance were included in the multivariate Cox regression analysis. Six variables were still identified as independent predictors of IAH (*p* < 0.05). The specific situation is shown in Table 2.

**Table 2:** Multi-factor analysis of IAH risk factors in ICU patients.

Factors	Regression coefficient (β)	Standard error (SE)	*P*	HR	95%CI
Pre-ICU surgery (yes)	0.421	0.178	0.018*	1.524	1.075–2.161
Abdominal distension (yes)	0.734	0.166	<0.001*	2.082	1.505–2.881
Increased abdominal cavity contents (yes)	0.619	0.165	<0.001*	1.857	1.343–2.567
Intraperitoneal fluid accumulation (yes)	0.405	0.170	0.017*	1.500	1.074–2.095
Shock (yes)	0.591	0.200	0.003*	1.806	1.218–2.677
APACHE II	0.046	0.015	0.002*	1.047	1.016–1.078

^*^ means *p* < 0.05 (statistically significant).

### 3.4 Construction of nomogram model

**Figure 2:**
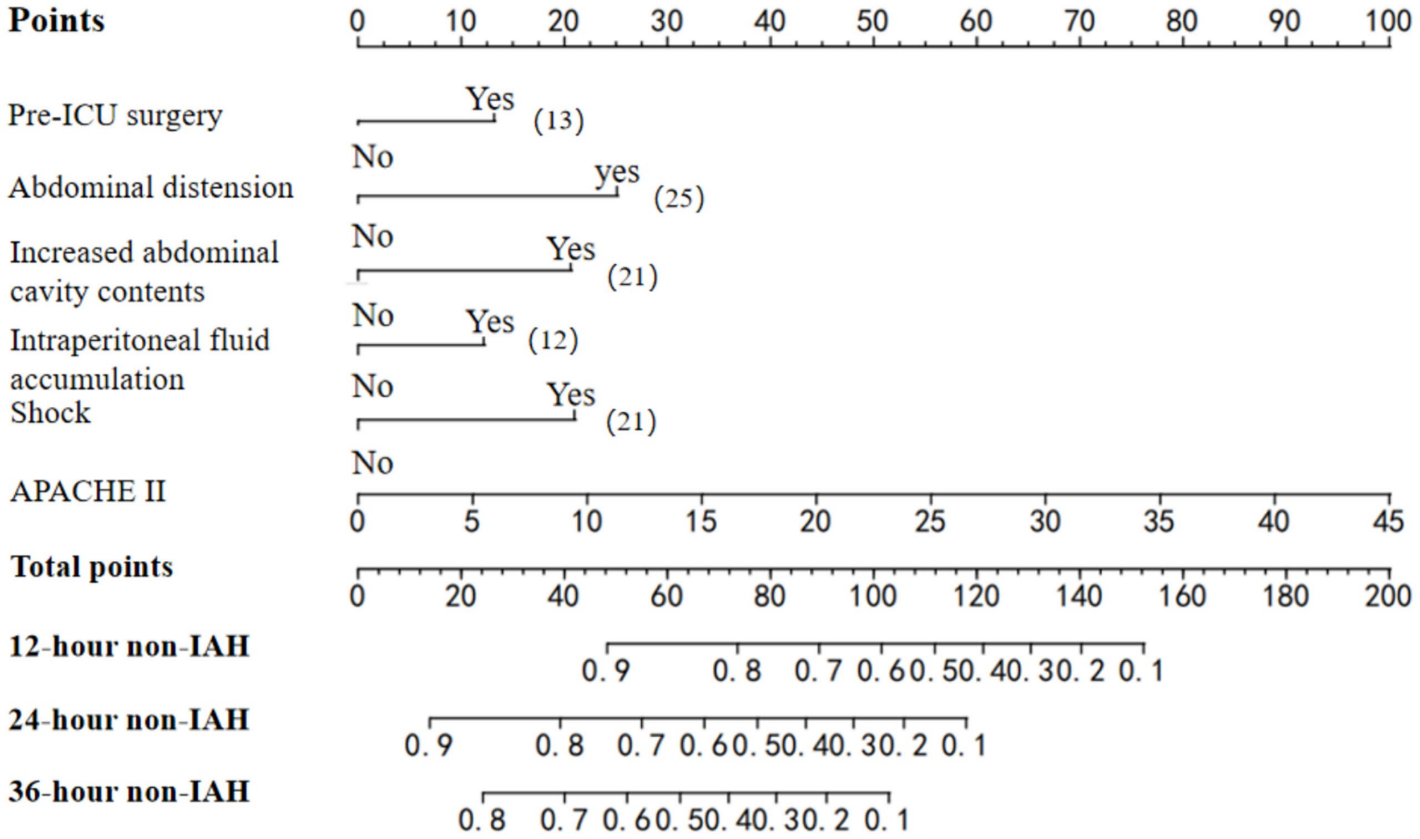
Risk prediction nomogram model for IAH in adult patients in the ICU.

Using the six independent predictors, we constructed a nomogram to estimate the probability of remaining IAH-free at 12, 24, and 36 h after ICU admission (Figure 2). To obtain the predicted IAH risk at a given time point, the user locates each predictor on the corresponding axis, draws a vertical line to the “Points” scale to assign a point value, sums the points, and then reads the predicted non-IAH probability from the “12 h Survival,” “24 h Survival,” or “36 h Survival” scales. The IAH risk is 1 minus this survival probability. For example, a patient with pre-ICU surgery (13 points), abdominal distension (25 points), Increased abdominal cavity contents (21 points), Intraperitoneal fluid accumulation (12 points), shock (21 points), and APACHE II score of 20 (44 points) would have a total of 136 points, corresponding to a 12 h IAH-free probability of approximately 0.24, 66% risk of IAH within 12 h.

### 3.5 Model performance and internal validation

The nomogram demonstrated excellent discrimination, with a C-index of 0.826 (95%CI: 0.736–0.890). After Bootstrap resampling (1,000 iterations), the bias-corrected C-index remained stable at 0.826 (95%CI: 0.792–0.861), indicating good internal validity.

Time-dependent AUCs were 0.836 (95%CI: 0.787–0.884) at 12 h, 0.932 (95%CI: 0.900–0.964) at 24 h, and 0.913 (95%CI: 0.877–0.950) at 36 h, indicating excellent predictive accuracy across all three time points. The optimal cut-off points (Youden index) and corresponding sensitivity/specificity were: at 12 h, cut-off = 112.7 points, sensitivity 64.5%, specificity 89.0%; at 24 h, cut-off = 93.1 points, sensitivity 80.8%, specificity 93.7%; at 36 h, cut-off = 78.6 points, sensitivity 82.3%, specificity 94.3%.

**Figure 3:**
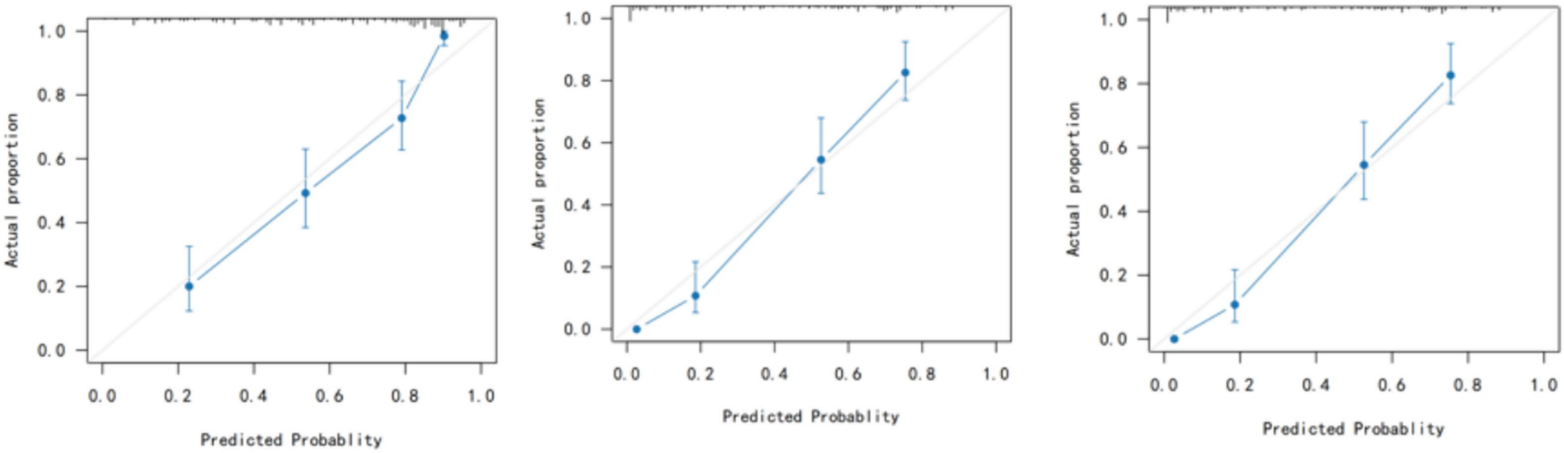
Calibration curves at 12 h, 24 h, and 36 h after admission to the ICU.

Calibration plots showed excellent agreement between predicted and observed IAH probabilities at 12, 24, and 36 h (Figure 3). The calibration curves were close to the ideal 45-degree line, with no evidence of systematic over- or under-prediction.

**Figure 4:**
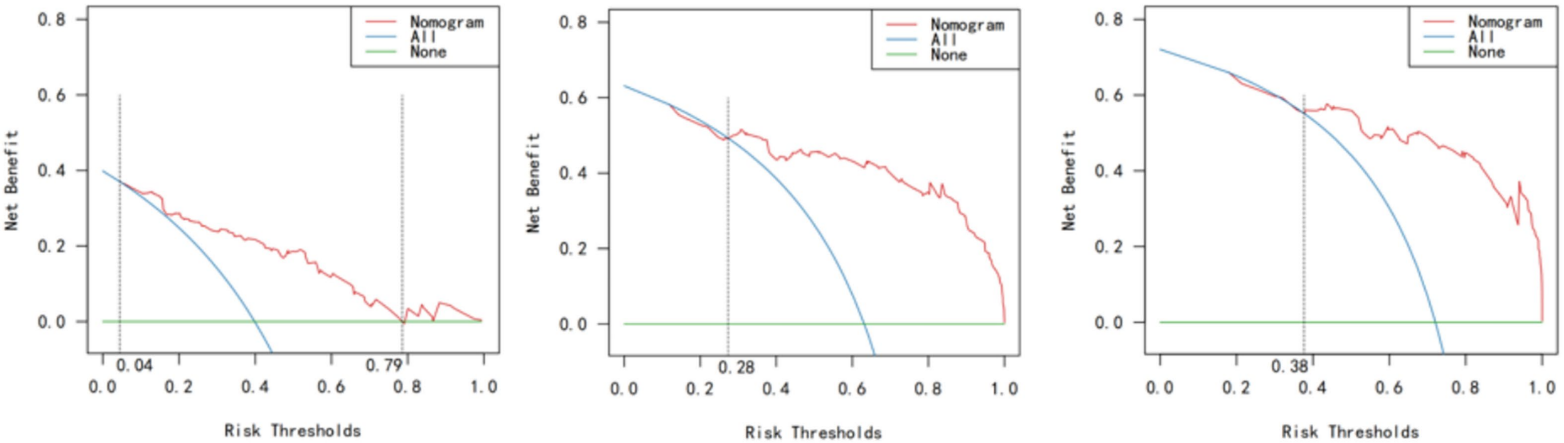
DCA curves at 12 h, 24 h, and 36 h after admission to the ICU.

Decision curve analysis (Figure 4) demonstrated that the nomogram provided a positive net benefit across a wide range of threshold probabilities. Specifically, at 12 h, the model was clinically useful for thresholds between 4 and 79%; at 24 h, for thresholds >28%; and at 36 h, for thresholds >38%. This indicates that using the nomogram to guide IAH monitoring and intervention would improve patient outcomes compared to either treating all patients or treating none. The DCA curve graph is shown in Figure 4.

## 4 Discussion

### 4.1 IAH is common and occurs early in ICU stay

This prospective study confirms that IAH is a frequent complication in critically ill patients, affecting three-quarters of our cohort. The incidence of 75.9% is higher than the 30–50% often cited in earlier reports (2, 4), which may reflect differences in case mix, monitoring frequency, or the use of more sensitive IAP measurement techniques. The high incidence of IAH is likely also due to the specific case mix of patients enrolled. Notably, the median onset of IAH was 12 h, with the vast majority of cases occurring within the first 36 h. This early window of vulnerability underscores the need for prompt risk stratification upon ICU admission. Delayed recognition can lead to progression to ACS, which carries mortality rates exceeding 50% (10). Therefore, a tool that identifies high-risk patients within the first day of ICU stay is of paramount clinical importance.

### 4.2 Independent risk factors

The six predictors retained in our final model align with the well-established pathophysiological triad of IAH: (1) increased intraluminal or intraperitoneal volume, (2) decreased abdominal wall compliance, and (3) capillary leak and fluid overload (5, 22).

#### 4.2.1 Pre-ICU surgery

Pre-ICU surgery emerged as a significant predictor, with a 1.524-fold increased hazard. Surgical patients, especially those undergoing gastrointestinal, hepatobiliary, or trauma procedures, are prone to postoperative ileus, intra-abdominal bleeding, tissue edema, and aggressive fluid resuscitation (23, 24). These factors collectively increase intra-abdominal volume and reduce compliance. Our finding corroborates studies showing that surgical patients have a higher IAP and IAH incidence compared to medical ICU patients (25, 26).

#### 4.2.2 Abdominal distension

Abdominal distension was the strongest predictor (HR = 2.08). While distension is a common clinical sign, it is often overlooked or attributed to other causes. In the context of IAH, abdominal distension reflects increased abdominal girth due to intraperitoneal or intraluminal accumulation (27). The WSACS guidelines recommend measuring IAP when abdominal distension is present, especially if accompanied by organ dysfunction (5). Our results provide quantitative evidence supporting this recommendation.

#### 4.2.3 Increased abdominal cavity contents

Increased abdominal cavity contents (HR = 1.86) encompass conditions such as gastroparesis, ileus, intestinal obstruction, and tumors. These conditions directly elevate IAP by occupying space within the abdominal cavity. Early interventions such as nasogastric decompression, prokinetic agents, or relief of obstruction can mitigate this risk (28).

#### 4.2.4 Intraperitoneal fluid accumulation

Intraperitoneal fluid accumulation (HR = 1.50) includes ascites, hemoperitoneum, and pneumoperitoneum. Fluid in the peritoneal cavity directly compresses viscera and elevates IAP. In patients with cirrhosis, ascites is a well-known cause of IAH, and large-volume paracentesis can rapidly reduce IAP (29, 30). Similarly, in trauma patients with hemoperitoneum, surgical evacuation may be necessary.

#### 4.2.5 Shock

Shock (HR = 1.81) is a critical driver of IAH through multiple mechanisms. Shock induces systemic inflammation and capillary leak, leading to fluid extravasation into the interstitium and abdominal cavity (31). Resuscitation with large volumes of crystalloids exacerbates this process, promoting visceral edema and further increasing IAP (32, 33). The relationship between shock, fluid resuscitation, and IAH has been documented extensively (34–36). Our findings reinforce the need for judicious fluid management in shock, with consideration of dynamic parameters and early use of vasopressors to limit fluid overload.

#### 4.2.6 APACHE II score

APACHE II score (HR = 1.047 per point) served as a composite marker of illness severity. Each one-point increase in APACHE II was associated with a 4.7% increase in IAH hazard. This aligns with studies showing that sicker patients are more likely to develop IAH (19). However, APACHE II alone is insufficient for IAH prediction, as it does not incorporate abdominal-specific variables. Our nomogram combines APACHE II with five abdominal and hemodynamic factors, thereby providing a more comprehensive risk assessment.

Several variables that were significant in univariable analysis did not remain in the multivariable model. Age, for example, lost significance after adjusting for APACHE II and other factors, suggesting that age-related risk is mediated by disease severity and comorbidities. Similarly, lactate and urea, while markers of tissue hypoxia and renal dysfunction, were likely captured by shock and APACHE II. The exclusion of these variables does not negate their clinical relevance; rather, it indicates that the six retained predictors are sufficient to explain the variation in IAH risk in our cohort.

### 4.3 Comparison with existing prediction models

To our knowledge, this is among the few nomograms developed specifically to predict intra-abdominal hypertension in a mixed medical surgical ICU population using exclusively bedside available clinical variables. Prior studies have largely focused on specific subgroups such as patients with acute pancreatitis (37) or trauma (38), or have identified risk factors without constructing a quantitative predictive tool. Our model addresses a broader ICU population with a practical point of care approach.

The Acute Physiology and Chronic Health Evaluation II (APACHE II) and Sequential Organ Failure Assessment (SOFA) scores are well established tools for assessing disease severity and organ dysfunction in critically ill patients. Although both scores correlate with IAH occurrence, they were designed to quantify overall illness severity rather than to specifically predict IAH risk (39). As generic indices, they lack abdominal specific pathophysiological variables, which limits their standalone discriminative ability. In our cohort, APACHE II score alone yielded a C index of approximately 0.68, markedly lower than the 0.826 achieved by our combined nomogram. This difference highlights the incremental value of incorporating abdominal and hemodynamic factors into a dedicated IAH risk model.

A published nomogram by Chen et al. (40) incorporated ultrasound derived parameters including right renal resistance index and right diaphragm thickening rate, combined with serum lactate, to predict IAH in critically ill patients. That model achieved an AUC of 0.956. Although it exhibits excellent predictive accuracy, its clinical application requires ultrasound equipment and trained personnel, which may not be uniformly available across all ICU settings. In contrast, our nomogram relies on six easily obtainable clinical variables: pre ICU surgery, abdominal distension, increased intra abdominal contents, intraperitoneal fluid accumulation, shock, and APACHE II score. These factors can be rapidly assessed at the bedside without specialized instrumentation or additional training, thereby enhancing the tool’s practicality and broader applicability.

By generating time dependent risk estimates at clinically relevant intervals of 12, 24, and 36 h post admission, our nomogram provides a simple bedside adjunct to clinical judgment. It helps clinicians tailor IAP monitoring frequency and implement timely preventive measures.

### 4.4 Clinical implications

From a nursing standpoint, the nomogram is particularly valuable. Bedside nurses are often the first to observe changes in abdominal girth, patient discomfort, or deviations in vital signs. With a simple point-based tool, they can quickly estimate IAH risk and alert the medical team. This empowers nurses to initiate early IAP monitoring and implement non-pharmacological interventions (41, 42).

For physicians, the nomogram aids in decision-making regarding fluid resuscitation, diuretic therapy, or surgical consultation. In patients with shock and high IAH risk, a more restrictive fluid strategy or early use of vasopressors may be considered. In those with intraperitoneal fluid accumulation, drainage procedures can be performed earlier (43). The nomogram also facilitates communication with the multidisciplinary team and with patients’ families, providing a visual representation of risk.

## 5 Conclusion

We developed and internally validated a nomogram incorporating six independent predictors—pre-ICU surgery, abdominal distension, Increased abdominal cavity contents, intraperitoneal fluid accumulation, shock, and APACHE II score, that accurately predicts the risk of IAH within the first 36 h of ICU admission. The model demonstrated excellent discrimination, calibration, and clinical utility. This simple bedside tool can empower clinicians, particularly nurses, to identify high-risk patients early, tailor IAP monitoring frequency, and implement targeted preventive strategies, ultimately improving outcomes in critically ill patients.

However, limitations must be acknowledged. No external validation cohort was included in this study; the model was evaluated using internal bootstrap resampling only. First, the study was conducted in two hospitals in a single Chinese province, which may limit applicability to other regions or healthcare systems. External validation in independent, multi-center cohorts is essential before widespread clinical adoption. Second, convenience sampling could introduce selection bias, though we attempted to enroll consecutive eligible patients. Third, we did not collect data on certain potential predictors, such as detailed fluid balance (cumulative fluid balance over time), type of surgery (laparoscopic vs. open), or specific ventilator settings, which might improve model performance. Fourth, the observation period was limited to 6 days; longer-term IAH risk was not assessed. Fifth, formal collinearity diagnostics were not performed prior to multivariable analysis. Sixth, once IAH was detected in a patient, further IAP measurements were not systematically recorded; therefore, the duration of IAH and its relationship to clinical outcomes could not be evaluated.

Future research should focus on external validation in diverse ICU populations, including medical, surgical, and trauma ICUs, and in different countries. Finally, qualitative research exploring nurses’ and physicians’ perceptions of the tool’s usability and impact on clinical workflow would be valuable for implementation.

## Data Availability

The datasets presented in this article are not readily available because data are not publicly available due to ethical restrictions and informed consent agreements that limit data use to the original research team only. Requests to access the datasets should be directed to Yanrui Tan, tanyr98@126.com.
